# Nightmare Disorder and Isolated Sleep Paralysis

**DOI:** 10.1007/s13311-020-00966-8

**Published:** 2020-11-23

**Authors:** Ambra Stefani, Birgit Högl

**Affiliations:** Department of Neurology, Medical University of Innsbruck (MUI), Anichstrasse 35, 6020 Innsbruck, Austria

**Keywords:** Dream, REM sleep, REM muscle atonia, polysomnography, parasomnia, REM sleep behavior disorder.

## Abstract

Nightmare disorder and recurrent isolated sleep paralysis are rapid eye movement (REM) parasomnias that cause significant distress to those who suffer from them. Nightmare disorder can cause insomnia due to fear of falling asleep through dread of nightmare occurrence. Hyperarousal and impaired fear extinction are involved in nightmare generation, as well as brain areas involved in emotion regulation. Nightmare disorder is particularly frequent in psychiatric disorders and posttraumatic stress disorder. Nonmedication treatment, in particular imagery rehearsal therapy, is especially effective. Isolated sleep paralysis is experienced at least once by up to 40% of the general population, whereas recurrence is less frequent. Isolated sleep paralysis can be accompanied by very intense and vivid hallucinations. Sleep paralysis represents a dissociated state, with persistence of REM atonia into wakefulness. Variations in circadian rhythm genes might be involved in their pathogenesis. Predisposing factors include sleep deprivation, irregular sleep–wake schedules, and jetlag. The most effective therapy consists of avoiding those factors.

**Supplementary Information:**

The online version contains supplementary material available at 10.1007/s13311-020-00966-8.

## Introduction

Nightmare disorder and recurrent isolated sleep paralysis (RISP) are both listed as rapid eye movement (REM) sleep-related parasomnias by the International Classification of Sleep Disorders 3rd Edition (ICSD-3) [1]: nightmare disorder occurs during REM sleep and RISP is a dissociated state in which REM sleep atonia continues into wakefulness. Both disorders are considered unpleasant experiences and cause clinically significant distress to those who suffer from them.

## Nightmare Disorder

### Diagnostic Criteria

Nightmare disorder is characterized by repeated nightmares. The dream content of these nightmares is remembered on awakening and can cause significant distress or impairment, including a subsequent fear of going back to sleep through fear that the nightmare may continue or re-occur. According to the ICSD-3 (Table 1), nightmare disorder consists of repeated occurrences of extended, extremely dysphoric, and well-remembered dreams that usually involve threats to survival, security, or physical integrity. On awakening from such dreams, the individual becomes alert quickly, but the dream experience and/or the sleep disturbance caused by the dysphoric dream cause clinically significant distress that can result in mood disturbance, social and cognitive impairments, and negative impacts on other areas of social, occupational, and educational functioning [1]. In addition to these characteristics, the American Psychiatric Association in its Diagnostic and Statistical Manual of Mental Disorders (DSM-5) also stipulates that a frequency criterion be met: nightmares should occur at least once a week for a positive diagnosis to be made [2].

**Table 1:** Diagnostic criteria for nightmare disorder according to the International Classification of Sleep Disorders, 3rd edition (ICSD-3) [1]

Criteria A–C must be met:	
A. Repeated occurrences of extended, extremely dysphoric, and well-remembered dreams that usually involve threats to survival, security, or physical integrity.	
B. On awakening from the dysphoric dreams, the person rapidly becomes oriented and alert.	
C. The dream experience, or the sleep disturbance produced by awakening from it, causes clinically significant distress or impairment in social, occupational, or other important areas of functioning as indicated by the report of at least one of the following: 1. Mood disturbance (e.g., persistence of nightmare affect, anxiety, dysphoria) 2. Sleep resistance (e.g., bedtime anxiety, fear of sleep/subsequent nightmares) 3. Cognitive impairments (e.g., intrusive nightmare imagery, impaired concentration, or memory) 4. Negative impact on caregiver or family functioning (e.g., nighttime disruption) 5. Behavioral problems (e.g., bedtime avoidance, fear of the dark) 6. Daytime sleepiness 7. Fatigue or low energy 8. Impaired occupational or educational function 9. Impaired interpersonal/social function	

Several medical conditions have been identified as being risk factors, including chronic disorders such as migraine, bronchitis, and asthma [3]. In addition, several classes of drugs (e.g., beta-blockers and some selective serotonin reuptake inhibitors [SSRIs]) and alcohol withdrawal may provoke nightmares, as these substances affect the structure of REM–nonREM cycles [4, 5]. There are conflicting data on the impact of low socioeconomic status on nightmare incidence [4]. Nightmares can also occur as part of the symptomatology of other sleep disorders, namely REM sleep behavior disorder (RBD), narcolepsy, trauma-associated sleep disturbance, or posttraumatic stress disorder (PTSD) [6, 7].

#### Evaluation of Nightmare Disorder

It is necessary to perform a detailed sleep history that should include questions about previous psychiatric disorders, as well as a history of medication and substance intake. Due to the frequent underdetection and underreporting of nightmares, it is important that sleep history taking includes at least one question about the occurrence of nightmares. It is important to determine if any other symptoms of parasomnias, e.g., sleepwalking, dream enactment behavior, or sleep paralysis are present. One should ask about excessive daytime sleepiness and cataplexy to rule out nightmares in the context of narcolepsy. Interaction between sleep quality and the occurrence of nightmares and insomnia symptoms predicting sleep paralysis is reviewed in Denis et al. [8]. Scales, such as the nightmare frequency questionnaire, a nightmare distress questionnaire, and others have also been used and are reviewed in Gieselmann et al. [9].

Posttraumatic nightmares should be differentiated from idiopathic nightmares. Posttraumatic nightmares have content and elicit emotions closely related to trauma and present with more severe arousal, awakenings, strong aggression, and more helplessness than idiopathic nightmares [9].

#### Polysomnography and Imaging in Nightmare Disorder

A polysomnographic confirmation of a nightmare disorder is most often not required but should be performed when there is a suspicion that nightmares occur in the context of RBD or narcolepsy. Polysomnographic studies in patients with nightmares or nightmare disorders have been extensively reviewed [6] and are characterized by more frequent awakenings [10] and alteration of sleep microstructure characteristics (increase in EEG alpha power, increased arousal-related phenomena in NREM–REM transitions, imbalance in sleep-promoting and arousing mechanisms) [11–13].

Functional magnetic resonance imaging (fMRI) studies show altered homogeneity in the cingulate cortex, right lower parietal lobe, and frontal gyri and other areas considered to be involved in the regulation of emotion or arousal modulation [14].

#### Children

It should be noted that nightmares in children and adolescents are particularly common, but they do not only affect the child but also usually affect at least one other family member who will have nonrestorative sleep because of the nightmares experienced by children [15]. As nightmares are frequent in children, a positive diagnosis requires all additional diagnostic criteria to be met [1].

### Prevalence

It is difficult to determine the true prevalence of nightmare disorder as the majority of epidemiological studies fail to distinguish it from nightmares [7]. There are, however, data on sporadic and frequent nightmares. Sporadic nightmares are a frequent phenomenon reported to affect 22% of the general adult population in Austria [16] and 36.2% of men and 45.1% of women in Finland [17]. The investigation of eight independent Finnish cross-sectional population surveys carried out between 1972 and 2007 has reported a prevalence of frequent nightmares in 2.9% of men and 4.4% of women. In adults, there is a slightly decreasing trend with age for women, but not for men who tend to have more frequent nightmares as they get older (war generations excluded) [17]. Nightmares are also reported to be more frequent in children than in adults [18] as well as in youth, with 14% of American public university students reporting frequent nightmares [19].

The prevalence of nightmares has been extensively studied in patients with psychiatric diseases [20–22]. A systematic review of 22 studies reported that the average prevalence of nightmare disorder was 38.9% in adults with a psychiatric disorder [23]. Nightmare disorder is prevalent in 66.7% of PTSD, 37.3% of mood disorders, 31.1% of personality disorders, and 15.6% of anxiety disorders [23]. Nightmare disorder has also been reported in patients with mixed psychiatric disorders, depression, and schizophrenia, with particularly elevated rates (> 30%) in the two latter conditions. In patients with psychiatric diseases, nightmares are reported to be frequent (several nights per week) and often cause multiple awakenings and difficulties getting back to sleep. Past trauma and high levels of stress were identified as both contributors and risk factors [22].

Another study reported that 24.9% of a sample of individuals with above-average psychopathology scores had clinically significant nightmare symptoms; however, 62.2% had not discussed their symptoms with a healthcare provider [20]. This indicates that nightmares are, in general, often underreported and, therefore, undetected [20]. It has been suggested that reasons for this underreporting are related to the belief that nightmares are untreatable; however, this was not found to be the case. The reporting of nightmare symptoms is positively correlated with symptom severity [20].

Finally, 35% of patients with inflammatory arthritis have been shown to meet criteria for REM sleep parasomnias [24], mainly due to probable RBD and nightmares (active nightmare disorder was present in 21% of the patients).

### Etiology and Pathophysiology

A recent study and consensus paper provides an extensive overview of current research into the etiology and treatment of nightmare disorders [9] and distinguishes between chronic nightmares and nightmare disorder in traumatized and nontraumatized individuals.

It is thought that chronic nightmares develop through the interaction of elevated hyperarousal and impaired fear extinction. Hyperarousal is important not only in nightmare disorder but also in insomnia and PTSD [9], two conditions in which nightmares are also frequent. Nightmare disorder might also involve disturbance of fear extinction, which should be enabled by normal sleep and dreaming in patients with nightmare disorders [9]. Both hyperarousal and impaired fear extinction, possibly facilitated by traumatic experiences, childhood adversity, and other trait susceptibilities, as well as physical and cognitive factors, contribute to the formation of a nightmare script, which is then thought to be replayed over and over and to generate the nightmare distress [10]. Posttraumatic nightmares are often specifically described as having a very stereotyped plot which replicates traumatic experiences [7].

Several factors have been suggested to facilitate hyperarousal and impaired fear extinction, including trait susceptibility (which may facilitate affect distress) and maladaptive beliefs [9], as the volitional attempt to suppress unwanted thoughts and feelings (thought suppression) have been reported to increase the likelihood that the suppressed thoughts re-occur in a person’s dreams [9]. Also, some physiological factors, such as sleep apnea, have been reported to play a role in the generation of nightmares [9], although this is controversial and has not been confirmed in all studies [25].

Additional theories have been advanced, one of which involves the mesocortical and mesolimbic dopamine system, as lesions involving this circuit have been associated with loss of dreaming [26].

Several models have been developed to explain the pathophysiology of nightmare disorder. These include a neurocognitive model by Levin and Nielsen [27], which proposes that nightmares reflect disturbed emotion regulation and involve the amygdala, medial, prefrontal cortex, hippocampus, and anterior cingulate cortex. There are conflicting data on the differential contribution of nightmare frequency on nightmare distress. A more recent study [28] suggested that similar brain areas are involved in disturbing dreams and the occurrence of distress during the day. An association has also been shown between nightmares and suicidal behavior, with poor emotion regulation being the possible link between the two [29].

### Treatment of Nightmare Disorder

Often underdetected and untreated, nightmare disorder can persist for decades. However, several medications and other nonmedication treatments are available for nightmare disorder. For nightmares associated with posttraumatic stress disorder, prazosin 1–3 mg at bedtime has been shown to be beneficial [5, 30] and is considered the first-line treatment. Prazosin has a long history of being used for the treatment of PTSD and PTSD-associated nightmares. It is a centrally acting alpha 1-adrenergic receptor antagonist. Raskind first observed its utility for PTSD-related nightmares [31] and it was then extensively used until unexpectedly large multicenter trials failed to show any effect of prazosin compared to placebo [32]. Since then, a position paper by the American Academy of Sleep Medicine (AASM) has highlighted a contradictory study [33] and downgraded its recommendation for prazosin, but still considers it to be the first-line pharmacological choice [34]. This is supported by a recent meta-analysis [35] confirming that prazosin does have a statistically significant benefit on PTSD symptoms and sleep disturbances.

Some nonmedication treatment approaches target the chronic repetition of nightmares, namely desensitization and exposure therapy or imagery rehearsal therapy (IRT), or in a few cases lucid dreaming. IRT is also recommended for PTSD-related nightmares [34] and has been successfully combined with cognitive–behavioral therapy [9]. IRT consists of encouraging the patient to write down the nightmare and then rewriting the end by giving it a more positive turn and dream outcome. The images for this more positive dream should be used during wakefulness during several 10–20-min daily sessions. IRT has also been shown to be beneficial in children [9]. Short or minimum contact intervention, through patient-applied Internet-based programs, have also been shown to be beneficial for nightmares.

A study investigating lucid dreaming as add-on therapy to Gestalt therapy in individuals with nightmare disorder reported positive results, with reduction of nightmare frequency, however, no differences were reported between those receiving Gestalt therapy alone and the group receiving Gestalt therapy and lucid dreaming. Interestingly, in the lucid dreaming group, dream recall frequency increased but at the same time, the nightmare frequency decreased [36].

Nonmedication treatment of nightmare disorder should also target maladaptive beliefs, which include ruminating and catastrophizing of nightmares and their consequences. Psychodynamic approaches focus on the particular meaning of a nightmare, but other treatment approaches do not take meaning into account [9].

One population that is particularly susceptible to PTSD and recurrent nightmares is refugees [9]. In refugees, the treatment of PTSD-related nightmares is often made difficult by the limited access to specific treatment and limited availability of interpreters, as well as due to the continued effect of nonverbalizable trauma, migration stress, uncertainty about asylum status, concerns about family members still unsafe, cultural and language difficulties, and perceived discrimination and racism [9].

Ultimately, the treatment approach depends on the individual patient and whether nightmare disorder is isolated or occurs in the context of PTSD or psychiatric diseases. The AASM recommendations for the treatment of nightmare disorder in adults are summarized in Table 2 [34].

**Table 2:** American Academy of Sleep Medicine (AASM) recommendations for the treatment of nightmare disorder in adults [34]

•PTSD-associated nightmares and nightmare disorder: Recommended: imagery rehearsal therapy	
•PTSD-associated nightmares: Cognitive–behavioral therapy; cognitive–behavioral therapy for insomnia; eye movement desensitization and reprocessing; exposure, relaxation, and rescripting therapy; the atypical antipsychotics olanzapine, risperidone, and aripiprazole; clonidine; cyproheptadine; fluvoxamine; gabapentin; nabilone; phenelzine; prazosin; topiramate; trazodone; and tricyclic antidepressants	
•Nightmare disorder: May be used: cognitive–behavioral therapy; exposure, relaxation, and rescripting therapy; hypnosis; lucid dreaming therapy; progressive deep muscle relaxation; sleep dynamic therapy; self-exposure therapy; systematic desensitization; testimony method; nitrazepam; prazosin; and triazolam Not recommended: clonazepam and venlafaxine	

## Sleep Paralysis

Due to its somewhat bizarre and sometimes dramatic clinical presentation as well as its frequent occurrence in the general population, sleep paralysis has often been portrayed in the arts, by many writers including Fyodor Dostoevsky and Guy de Maupassant [37, 38] and by artists such Heinrich Füssli [39]. There are mentions of sleep paralysis that date back to medieval Persia [40].

### Diagnostic Criteria

Sleep paralysis not occurring in association with narcolepsy is defined as isolated sleep paralysis. RISP is defined by the ICSD-3 (Table 3) as a recurrent inability to move the trunk and all the limbs at sleep onset (hypnagogic form) or upon awakening from sleep (hypnopompic form). Each RISP episode can last from a few seconds to a few minutes and causes clinically significant distress including bedtime anxiety or fear of sleep. The episodes resolve spontaneously or upon sensory stimulation, e.g., when someone touches the person or an alarm clock sounds, or sometimes also by subject-initiated movements of the eyes. RISP should not be better explained by another sleep disorder (especially narcolepsy), mental disorder, medical condition, medication, or substance use.

**Table 3:** Diagnostic Criteria for Recurrent Isolated Sleep Paralysis according to the International Classification of Sleep Disorders, 3rd edition (ICSD-3) [1]

Criteria A–D must be met:	
A. Recurrent inability to move the trunk and all of the limbs at sleep onset or upon awakening from sleep.	
B. Each episode lasts seconds to a few minutes.	
C. The episodes cause clinically significant distress including bedtime anxiety or fear of sleep.	
D. The disturbance is not better explained by another sleep disorder (especially narcolepsy), mental disorder, medical condition, medication, or substance use.	

The first-ever occurrence of sleep paralysis can be very frightening due to the mixture of subjective wakefulness and complete inability to move (if individuals only experience very slow movement or feel as if their body is heavier than usual, this is not sleep paralysis) combined with subjective awakenings. In addition, because not only the striated limb muscles are affected but also the auxiliary respiratory muscles (intercostal muscles), and the diaphragm is the only respiratory muscle not affected during the episodes, patients during sleep paralysis often experience some oppression/pressure on the chest or a heavy weight on the rib cage.

RISP can be accompanied by very intense and vivid hallucinatory experience, which may be visual, tactile, or auditory, or the sense of a presence in the room. RISP is a benign phenomenon. Predisposing factors include sleep deprivation and jetlag [41]. Therefore, all patients with sleep paralysis should be screened for sleep quality and insomnia symptoms [8].

#### Differential Diagnosis

Differential diagnosis of RISP includes (in particular when associated with hallucinations) sleep terrors, nightmare disorder, lucid dreaming, and more rarely RBD [42]. Moreover, other causes need to be considered: any paralysis upon awakening should also be differentiated from wake-up stroke; hypokalemic paralysis can occur during day and night but also upon awakening from sleep.

It is not always easy to distinguish nightmares from sleep-onset or sleep-offset hallucinations in patients with sleep paralysis, but both are recognized and can even co-occur in a single patient. Frequent nightmares are associated with sleep disruption, insomnia, impaired daytime functioning, and other mental complaints [9].

Patients with nightmares or nightmare disorder awaken completely, are quickly alert, and remember the dream content. In contrast to nightmare disorder, night terrors present with confusional arousals and incomplete awakenings, and in children, there are often difficulties in being comforted [9].

In a retrospective study, lucid dreaming was associated positively with sleep paralysis featuring intense vestibulo-motor hallucinations (as opposed to intruder and incubus hallucinations). The authors suggested therefore that both experiences are REM dissociated states characterized by positive emotion [42].

In RBD, dreams are often violent, but not always nightmares [43]. In particular, in patients with Parkinson’s disease, no major differences between patients with and without RBD in the action-filledness, vividness, or threat content of dreams have been reported. However, dreams were more often negatively than positively toned in Parkinson’s disease patients with RBD [44]. The most often reported dreams in RBD involve counterattacking a human or animal assault, whereas in sleep terrors fleeing a disaster is more often reported [45].

### Prevalence

Most prevalence studies suggest that 15–40% of the population of younger individuals have experienced at least one episode of sleep paralysis, but there are also a few studies reporting a much lower prevalence, ~ 5% or 6% [1]. Sleep deprivation, irregular sleep–wake schedules, or jetlag have been identified as predisposing factors, and it has been shown that sleep paralysis episodes are more common in the supine position [1].

### Etiology and Pathophysiology

Sleep paralysis has been listed among the dissociated states [46]. An early study elicited sleep paralysis by sleep interruption and concluded that sleep paralysis occurs in the transition between REM and wakefulness and during ambiguous REM sleep, in contrast to lucid dreaming, which occurred during unequivocal REM sleep [47]. In particular, this study showed that during polysomnography (PSG) EEG, there was a typical sequence, with the intrusion of an alpha EEG into REM sleep, followed by an arousal response, and then the persistence of REM atonia into wakefulness [47]. Moreover, many sleep paralysis episodes occurred from sleep-onset REM periods (SOREMP); thus, the authors suggested that conditions inducing SOREMP, such as disruption of the circadian sleep–wake rhythm or interruption of the REM–NREM cycle might favor sleep paralysis.

For a review on further historic and more recent studies into the neurophysiology of sleep paralysis, see Stefani et al. [6]). For the co-occurrence of sleep paralysis together with hypnagogic or hypnopompic hallucinations, a three-factor structural model (intruder, incubus, and unusual bodily experience) has been proposed [48]. Intruder consists of sensed presence, fear, and auditory and visual hallucinations. Incubus comprises pressure on the chest, breathing difficulties, and pain. Unusual bodily experiences consist of floating/flying sensations, out-of-body experiences, and feelings of bliss, related to physically impossible experiences generated by conflicts of endogenous and exogenous activation related to body position, orientation, and movement [41].

Few families with RISP have been reported [1]. First, genetic analysis points to impaired circadian rhythm, as variations in the circadian rhythm gene PER2 have been shown to increase the odds of sleep paralysis [8]. However, genetic studies for RISP are lacking, and genome-wide association studies have not been performed.

### Treatment

RISP is usually self-limiting and does not impair daytime function. As sleep paralysis is facilitated by chronic sleep deprivation or an irregular sleep–wake schedule, it is important to inform patients about sleep hygiene and the necessity of obtaining sufficient sleep (7–9 h per night) and adhering to regular sleep–wake schedules, whenever possible. Theoretically, because sleep paralysis occurs most frequently in the supine position, patients may be advised to sleep in a lateral or prone position after travel across multiple time zones. The occurrence of sleep paralysis during sleep in sitting positions (such as in airplanes) has not been reported.

If patients have bed partners, they may be instructed to touch the subject when low vocalizations occur in the morning before awakening.

Drug treatment will rarely be necessary for isolated sleep paralysis, but it has been reported that in patients with narcolepsy, tricyclic or other antidepressants do not only improve cataplexy but also reduce the number of sleep paralysis episodes. Cognitive–behavioral therapy for sleep paralysis includes coping strategies for frightening, hallucinations, or the imaginative replay of previous successful attempts to resolve the sleep paralysis [6].

## Conclusions

The REM parasomnias nightmare disorder and recurrent isolated sleep paralysis can be a cause of significant distress.

Insomnia is a frequent consequence of nightmare disorder, as patients are frightened of nightmare occurrence and, therefore, fear falling asleep. Brain areas responsible for emotion regulation are involved in nightmare generation, as well as hyperarousal and impaired fear extinction. Conditions in which nightmare disorder is particularly frequent include psychiatric disorders and posttraumatic stress disorder. Nonmedication treatment, in particular imagery rehearsal therapy, is particularly effective for this condition.

Isolated sleep paralysis is a common condition that is experienced at least once by up to 40% of the general population, whereas recurrence is less frequent. Very intense and vivid hallucinations can accompany the sleep paralysis episodes. It represents a dissociated state, with persistence of REM atonia into wakefulness. Familial cases have been reported, and the pathogenesis of sleep paralysis may involve variations in circadian rhythm genes. Predisposing factors include sleep deprivation, irregular sleep–wake schedules, and jetlag. Avoiding these factors is the most effective therapy.

## Supplementary Information

ESM 1 (PDF 1224 kb)
